# Glioblastoma Cells Do Not Affect Axitinib-Dependent Senescence of HUVECs in a Transwell Coculture Model

**DOI:** 10.3390/ijms21041490

**Published:** 2020-02-21

**Authors:** Matilde Merolle, Maria Patrizia Mongiardi, Maurizia Piras, Andrea Levi, Maria Laura Falchetti

**Affiliations:** CNR-IBBC, Institute of Biochemistry and Cell Biology, 00015-Monterototondo Scalo-Rome, Italy; MatildeMerolle@live.it (M.M.); mariapatrizia.mongiardi@cnr.it (M.P.M.); pirasmaurizia@gmail.com (M.P.); andrea.levi@cnr.it (A.L.)

**Keywords:** Axitinib, endothelial cells, glioblastoma, senescence

## Abstract

Axitinib is an orally available inhibitor of tyrosine kinases, with high specificity for vascular endothelial growth factor receptors (VEGFRs) 1, 2, and 3. It is approved for the treatment of advanced renal cell carcinoma and is in phase II clinical trials for recurrent glioblastoma (GBM). GBM is a brain tumor peculiar in its ability to induce neoangiogenesis. Since both GBM tumor cells and endothelial cells of tumor vasculature express VEGFRs, Axitinib exerts its inhibitory action on both tumor and endothelial cells. We and others previously demonstrated that Axitinib triggers cellular senescence. In particular, Axitinib-dependent senescence of HUVECs (human umbilical vein endothelial cells) is accompanied by intracellular reactive oxygen species(ROS) increase and early ataxia telangiectasia mutated(ATM) activation. Here we wondered if the presence of glioblastoma tumor cells could affect the HUVEC senescence upon Axitinib exposure. To address this issue, we cocultured HUVECs together with GBM tumor cells in transwell plates. HUVEC senescence did not result in being affected by GBM cells, neither in terms of β galactosidase activity nor of proliferation index or ATM phosphorylation. Conversely, Axitinib modulation of HUVEC gene expression was altered by cocultured GBM cells. These data demonstrate that the GBM secretome modifies HUVECs’ transcriptomic profile upon Axitinib exposure, but does not prevent drug-induced senescence.

## 1. Introduction

Axitinib is an orally available inhibitor of tyrosine kinases, with high specificity for vascular endothelial growth factor receptors (VEGFRs) 1, 2, and 3 [1]. It is a small molecule (MW 386.74) and its IC50 for the VEGF family of receptors is 10-fold lower than the ones of other tyrosine kinase inhibitors such as pazopanib, sunitinib, or sorafenib. The drug is approved for renal cell carcinoma (RCC) management by US FDA, EMA, UK MHRA, and Australian TGA. Axitinib clinical trials have been performed on children and adolescents with recurrent or refractory solid tumors [2], in neuroendocrine neoplasms [3], in metastatic breast cancer [4], in metastatic melanoma [5], and in recurrent glioblastoma (GBM) [6]. At the dosage employed in vivo on patients [7], it is possible that Axitinib efficacy depends on its ability to affect multiple kinases. In vitro, Axitinib selectively blocks VEGF-stimulated receptor autophosphorylation leading to inhibition of endothelial cell proliferation, survival, migration, and tube formation; in vivo, it blocks the growth and angiogenesis of human tumor xenografts in mice [1]. Recently, in vitro experiments showed that chronic Axitinib treatment induces senescence in glioblastoma cell lines [8]. Further, we demonstrated that a transient Axitinib pulse triggers cell senescence in the glioblastoma cell line U87MG and in the primary endothelial cellsHUVECs (human umbilical vein endothelial cells) [9]. Axitinib affects the gene expression profile and a change in the senescence-associated secretory phenotype (SASP) was observed upon Axitinib exposure under normoxic as well as hypoxic conditions [9,10].

Cell senescence triggered by therapies (either chemotherapy or radiation) is a critical side effect when treating tumor patients, since it promotes adverse collateral effects and cancer relapse [11]. Senescent cells’ influence on the tumor microenvironment is deep, and the result of an anticancer drug treatment is substantially affected by the senescent cell secretome. The role of SASP on the tumor microenvironment is a crucial issue when considering a drug’s side effects.

We previously addressed an Axitinib prosenescence activity on GBM tumor cells and on endothelial cells in culture. Here we wondered if in an in vitro system mimicking the in vivo interaction between tumor and endothelial cells, the presence of tumor cells affects Axitinib-triggered senescence of HUVECs.

## 2. Results

### 2.1. GBM Tumor Cells Do Not Affect Axitinib-Dependent Expression of SA-β-Galactosidase in HUVECs

**Figure 1:**
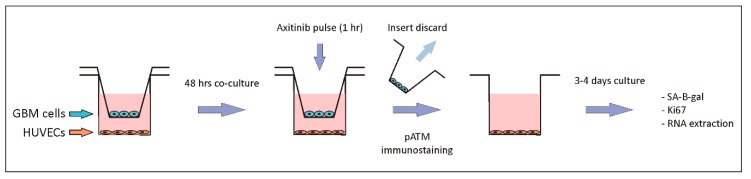
Schematic representation of the experimental model. The diagram summarizes the organization of the experiments performed in the paper.

The first question to be answered was if GBM tumor cells affect Axitinib-dependent senescence of HUVECs. To address this point, we cocultured HUVECs together with GBM cells using commercial transwells, where the two cell populations were divided by a polycarbonate membrane with a 0.4 µm pore size (Figure 1). The two cell populations plated in transwells had no physical contact and were not allowed to cross the transwell membrane, but shared the culture medium and reciprocally talked via their secreted factors. We used two different GBM cell lines, U87MG and A172, both expressing VEGF and its receptors, VEGFR1 and VEGFR2 [12]. HUVECs were plated in the well and GBM cells were plated in the removable insert. We used a mixed cell culture medium, made of a 1:1 ratio of endothelial and of GBM cells medium (see Materials and Methods section for details). 

After 48 h of coculturing, cells were exposed to a brief pulse (1 h) of 25 µM Axitinib. Using this protocol, we previously found that single cultures of HUVECs and of U87MG undergo senescence [10]. After the Axitinib pulse, the insert containing GBM cells was removed, and HUVECs were cultured in 100% endothelial cells growth medium for up to four days, fixed, and stained for acidic β-galactosidase activity, the gold standard marker for senescent cells (Figure 1). 

**Figure 2:**
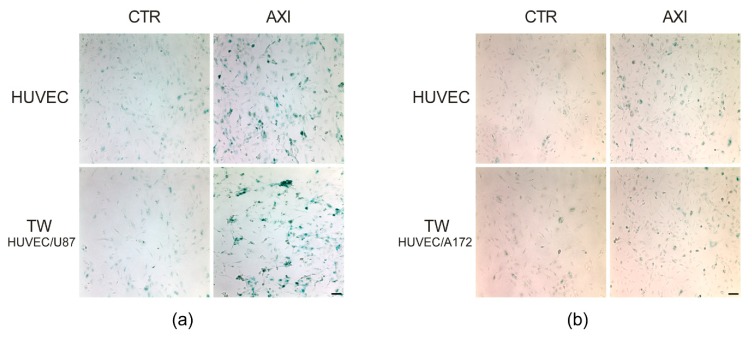
Axitinib-dependent SA-β- galactosidase staining of HUVECs wasnot altered by GBM cells coculture. Four days after Axitinib pulse, HUVECs appeared positive to β-galactosidase staining, with a typical blue color. This effect was not altered in HUVECs cocultured for 48 h in transwell with GBM cell lines, U87MG (**a**) or A172 (**b**). *n* = three biological replicates. Magnification 10×, scale bar 100 µm.

As a reference, we stained HUVECs cultured as single population under the same experimental setting of cocultures. Figure 2 shows β-galactosidase staining at four days following Axitinib treatment. We did not observe significant differences in the pattern of β-galactosidase-positive cells between HUVECs and HUVECs cocultured with U87 MG (Figure 2a) or A172 (Figure 2b).

### 2.2. GBM Tumor Cells Do Not Affect Axitinib-Dependent Ki-67 Expression in HUVECs

**Figure 3:**
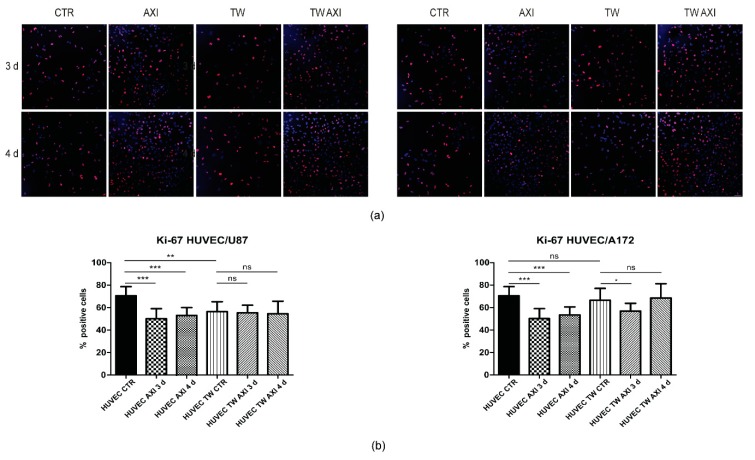
Proliferation rate of Axitinib-treated HUVECs was not affected by coculture with GBM cells. Ki-67 immunostaining was performed on control (sham-treated) HUVECs, either cultured alone or in transwell with U87MG (**a**,**b**, left panel) or A172 (**a**,**b**, right panel) GBM cells. Control cells, either single or transwell cultures, were fixed after 48 h of culturing. Axitinib-treated cultures were fixed three or four days following Axitinib pulse, as schematized in Figure 1. Mean values and standard deviation were generated from at least three biological replicates. Magnification 20×, scale bar 50 µm.

We then addressed HUVECs’ proliferation index by immunostaining with Ki-67 antibody (Figure 3a,b). Again, we cocultured for 48 h HUVECs with GBM cells, U87MG, or A172, exposed cells to the Axitinib pulse, and measured the percentage of Ki-67-positive cells three and four days post Axitinib treatment (Figure 1). As expected from previous results [9], the proliferation index of HUVECs significantly decreased following Axitinib exposure. In cocultures with U87MG, HUVECs reduced their proliferation rate and no further reduction was observed after Axitinib exposure. Conversely, in cocultures with A172, no significant difference between Ki-67 positivity of single and of cocultured HUVECs was found. Axitinib reduced HUVECs’ proliferation rate, although with a certain degree of variability (Figure 3b).

### 2.3. GBM Tumor Cells Do Not Affect Axitinib-Dependent Activation of ATM in HUVECs

**Figure 4:**
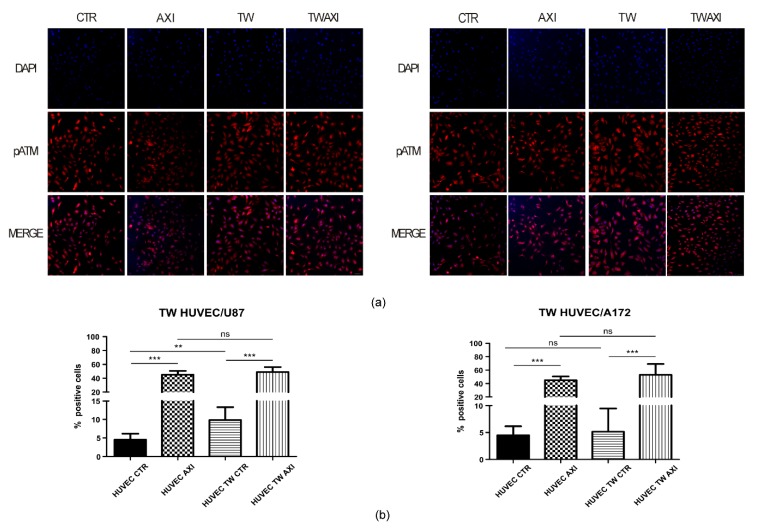
Axitinib-dependent ATM phosphorylation in HUVECs was not altered by GBM cells coculture. pATM immunostaining was performed on HUVECs cocultured with U87 (**a**,**b**, left panel) or A172 (**a**,**b**, right panel) GBM cells. CTR: sham-treated HUVECs; AXI: Axitinib-treated HUVECs; TW: sham-treated HUVECs cocultured in transwell with GBM cells for 48 h; TW AXI: HUVECs cocultured in transwell with GBM cells and treated with Axitinib. Immunofluorescence was performed at the end of the 1h Axitinib pulse. Mean values and standard deviation were generated from at least three biological replicates. Magnification: 20×; scale bar 50 µm.

ATM (ataxia telangiectasia mutated) kinase plays a key role in establishing and maintaining senescence. Although the well-addressed role for ATM in triggering cell senescence resides in promoting DNA damage response (DDR) following a genotoxic insult, we showed ATM involvement in Axitinib-driven senescence of HUVECs [9].We therefore wondered if GBM cells could interfere with Axitinib-dependent activation of ATM in cocultured HUVECs. To address this point, we cocultured HUVECs and GBM cells, either U87MG or A172, in transwell plates for 48 hours, as described above, and performed an immunofluorescence using an antibody targeting the active, phosphorylated form of ATM (pATM, phosphorylated serine 1981). Since we previously characterized that ATM activation follows Axitinib exposure as an early event, we fixed cells at the end of the one-hour Axitinib pulse (Figure 1). Figure 4a shows pATM staining upon Axitinib treatment. No difference in the staining pattern of pATM was apparent between single culture HUVECs and HUVECs cocultured with U87MG (left panel) or A172 (right panel) GBM cells. The percentage of pATM-positive HUVECs did not significantly differ between the two experimental groups of Axitinib-treated HUVECs (single culture vs. cocultures) (Figure 4b). Interestingly, we observed an increase of pATM in HUVECs cocultured with U87MG (4.18% and 10.11% in single and cocultured HUVECs, respectively; Student’s t-test, *p* < 0.01). It is reasonable to hypothesize that the presence of U87MG cells with a high proliferation rate, together with angiogenic-secreted factors, contribute to ROS increase in cocultured HUVECs. The different behavior in A172 cocultures might depend on the known heterogeneity of GBM cell lines.

### 2.4. GBM Tumor Cells Affect Axitinib-Dependent Gene Expression Profile of HUVECs

**Figure 5:**
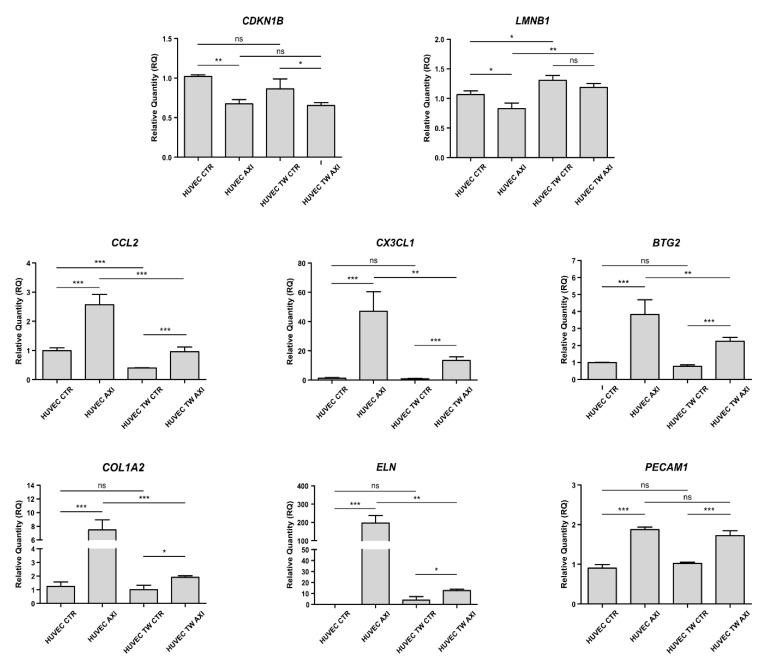
Axitinib- dependent modulation of HUVEC gene expression was altered by GBM coculture. Real-time PCR was performed on RNA extracted from HUVECs cultured alone or in transwell with U87MG, three days after Axitinib pulse. Senescence-related genes (CDKN1B and LMNB1), SASP genes (CCL2 and CX3CL1), extracellular matrix genes (COL1A2 and ELN), and an endothelial cell marker (PECAM1) expression were analyzed. Except for PECAM1, Axitinib-dependent mRNA modulation appeared altered in coculture system. Relative quantities were calculated normalizing for TBP and are given relative to control HUVECs (sham-treated). Mean values and standard deviation were generated from at least three biological replicates. * *p* value < 0.05; ** *p* value < 0.01; *** *p* value < 0.001.

We then wondered if the GBM cell secretome, which does not affect HUVECs’ entrance in cell senescence following Axitinib pulse, could instead affect Axitinib-mediated gene expression regulation of cocultured HUVECs. Again, we cocultured HUVECs together with U87MG in a transwell for 48 hours, as described above. Following a one-hour pulse with 25 µM Axitinib, we discarded the U87MG-containing transwell insert. HUVECs were cultured for a further three days to allow cells to acquire the senescent phenotype, before total RNA extraction and reverse transcription (Figure 1).We measured the expression of a pool of genes whose expression was modulated following Axitinib treatment [9] (Figure 5). Among these, we selected two genes regarded as markers of senescence, CDKN1B and LMNB1, encoding for p27 and Lamin B1, respectively [13]. Moreover, we evaluated expression changes of BTG2, a regulator of cell cycle progression, of the SASP genes CCL2 and CX3CL1, of the extracellular matrix components collagen type I α2 and elastin, encoded by COL1A2 and ELN genes, respectively, and of the endothelial cells’ phenotypic marker CD31, encoded by the PECAM1 gene, whose expression is regulated by Axitinib [9]. The expected p27 downregulation, following Axitinib-induced senescence, was not affected by the presence of U87MG. Conversely, in the transwell, LMNB1 expression was upregulated in HUVECs by U87MG, and Axitinib failed to significantly downregulate LMNB1 (although a decreasing trend was observed), likely because it does not suffice to blunt the upregulation induced by tumor cells per se, and irrespectively of senescence establishment.

Overall, the Axitinib-dependent gene expression change of the SASP genes CCL2 and CX3CL1, of the cell-cycle regulator BTG2, of the extracellular matrix components COL1A2 and ELN exhibited the same course. It was reduced in cocultures, when compared to single cultures, although a significant variation of their expression was retained (Figure 5). Finally, Axitinib-dependent PECAM1 induction was not affected by coculture with U87MG.

## 3. Discussion

Cell senescence is a cellular program that enforces a permanent cell cycle arrest to cells exposed to a variety of strong stresses [14]. Although not dividing, senescent cells are metabolically active and secrete proinflammatory cytokines, chemokines, growth factors, and extracellular matrix-remodeling proteins, collectively named senescence-associated secretory phenotype (SASP) [15]. Cell senescence is associated with physiological processes, such as tissue remodeling during embryo differentiation and wound healing, but it also plays an important role in organismal aging. Senescence has been thoroughly investigated in the context of neoplastic transformation, since it is an important cell autonomous tumor suppressor mechanism, which prevents propagation of damaged, potentially dangerous cancer cells. Through the activity of SASP, senescent cells may either promote [16] or suppress [17] cancer growth in a non-cell-autonomous way. Despite its importance in many physiological and pathological processes, senescence still lacks a precise definition, and a “senescence signature” is still an ongoing quest [18]. Generally, employed markers of cell senescence include SA-β-galactosidase, reduced Ki-67 expression, high expression of the cyclin-dependent kinase inhibitor p16INK4, and SASP. Different cellular insults result in senescent cells which may differ in their SASP [19], and, in turn, SASP may evolve over time [20]. Consequently, cell senescence is a very dynamic and complex process, especially when it involves distinct cell populations, which may reciprocally affect each other through their SASPs. In the present study, we purposely employed an experimental protocol which allowed us to study whether factors released by GBM cells alter the process through which Axitinib triggers senescence in HUVECs. In this experimental setting, acquisition of the senescent phenotype by HUVECs occurred in the absence of GBM cells, thus avoiding confounding effects due to two coevolving cell types. 

In a first set of experiments, we investigated whether preliminary coculture with GBM cells interferes with expression of SA-β-galactosidase by Axitinib-exposed HUVECs. Neither U87MG nor A172 GBM cells affected the expression of SA-β-galactosidase by HUVEC cells four days after Axitinib pulse, as shown in Figure 1. Similarly, the decreased expression of Ki-67, which characterizes non dividing cells, did not differ in control vs. transwell-cultured HUVECs upon treatment with Axitinib, as shown in Figure 3. Of note, we observed a small but reproducible decrease in the percentage of Ki-67-positive cells between control and transwell-cultured HUVECs in the absence of treatment with Axitinib. This decrease was significant in cocultures with U87MG but not in A172 co cultures, and may be due to consumption of nutrients by the rapidly proliferating U87MG cells, compared to A172 cells. It was previously characterized that U87MG and A172 GBM cell strains have different behavior in vitro and in vivo [21]. 

We recently demonstrated that Axitinib-dependent HUVEC senescence required production of reactive oxygen species (ROS) and an early activation of the ataxia telangiectasia mutated (ATM) kinase [9]. Here, quantification of active ATM after Axitinib treatment also failed to highlight differences between control and transwell-cultured HUVECs, as shown in Figure 4b. Interestingly, we observed an increase of activated ATM in HUVECs cocultured with U87MG for 48 h. The involvement of ATM in pathological angiogenesis has been previously described, as well as its role as a defender in response to ROS increase [22]. U87MG cells, which are highly proliferating and secrete high amount of angiogenic factors, possibly contribute to ROS increase in cocultured HUVECs, an effect probably reduced in the less tumorigenic A172 GBM. 

Finally, we quantified the expression of mRNAs of selected genes whose expression is altered in HUVECs that became senescent upon Axitinib treatment. These experiments are shown in Figure 5. We chose two genes which, according to our previous analysis [9], were downregulated in Axitinib-induced senescence (LMNB1 and CDKN1B), and genes which were upregulated (ELN, CX3CL1, BTG2, CCL2, COL1A2, and PECAM1). As far as downregulated genes were concerned, coculturing with GBM decreased the differences between control and Axitinib-treated HUVECs, in the case of LMNB1 likely because of its higher expression in transwell controls, and in the case of CDKN1B because of its lower expression in controls grown in the presence of GBM. 

Quite different was the response of upregulated genes whose response to Axitinib was significantly blunted in HUVEC cells cocultured with U87MG. Remarkably, since the RNAs were extracted from HUVEC cells four days after the Axitinib pulse, exposure to the GBM secretome appeared to induce long-lasting effects on the gene expression profile of HUVEC cells. This suggests that the GBM secretome may alter epigenetic marks in HUVEC cells. It is also possible that, as indicated by the decrease of Ki-67 and increase of active ATM, coculture with GBM represents a stress which blunts the response of HUVEC cells to a stronger stress, like the Axitinib pulse, a typical hormetic cellular response [23].

A last consideration concerns the biological significance of the reduced Axitinib-dependent expression of genes like CCL2, CX3CL1, COL1A2, and ELN in transwell-grown HUVEC cells. All these genes encode for proteins that may affect the tumor microenvironment. It appears as if the GBM secretome tends to flatten the changes in gene expression occurring in endothelial cells upon stresses, thus potentially decreasing the effect that senescent endothelial cells may exert on GBM. Future studies with a deeper characterization of GBM-dependent changes in endothelial gene expression profiles will help to clarify this point.

## 4. Materials and Methods

### 4.1. Cell Cultures and Chemicals

HUVECs (pooled donors) were purchased from ThermoFisher Scientific (Waltham, MA, USA), and grown in complete endothelial cell growth medium (EndoPan 3 Kit, Pan Biotech, Aidenbach, Germany). For the presented experiments, we used HUVECs between passage two and six. U87MG and A172 cells were purchased from ATCC and cultured in DMEM 4.5 g/L glucose (ThermoFisher Scientific, Waltham, MA, USA), supplemented with 10% fetal bovine serum (ThermoFisher Scientific).

Cells were grown at 37 °C in a humidified atmosphere of 5% CO_2_–95% air. All cell lines were regularly checked to exclude mycoplasma contamination by Mycoalert Detection Kit (Lonza, Basel, Switzerland).

Cocultures of HUVEC and U87MG or A172 cells were performed using transwell cell culture plates (Corning, Corning, USA) with a polycarbonate membrane insert (0.4 µm pore size). Briefly, HUVECs and GBM cells were plated in the well and in the insert, respectively, in EndoPan 3/DMEM at 1:1 ratio. Forty-eight hours post-plating, cells were treated with a pulse of 25 µM Axitinib for 1 h. After extensive washings, GBM-cell-containing inserts were removed, and HUVECs were cultured in 100% complete EndoPan 3 medium for up to four days. 

All chemicals were purchased from Sigma Aldrich (St. Louis, MO, USA). Axitinib and doxorubicin were resuspended in DMSO.

### 4.2. SA-β-Galactosidase Assay

The assay was performed according to the protocol by Dimri and colleagues [24]. Blue cells were visualized in bright field microscopy, acquired by MoticImagesPlus 3.0 (Motic, Hong Kong, China). 

### 4.3. Immunofluorescence

HUVECs grown on glass coverslips were fixed with 4% paraformaldehyde, permeabilized in 0.25% Triton X-100 for 5 min, fixed with ice-cold methanol for 10 min, and blocked with a solution of 3% BSA and 0.05% Tween20, for 30 min at 37 °C. After washing, cells were incubated in PBS containing 3% BSA and 0.05% Tween20, for 2 h at 37 °C, with primary antibodies: mouse Phospho ATM (Serine 1981) Mab (1:200; Cell Signaling, Danvers, MA, USA #4526), and rabbit anti Ki-67 (1:200; ThermoFisher Scientific#RM-9106-S1 Waltham, MA, USA). Cells were washed and incubated in 0.05% Tween 20, 1% BSA, and Alexa Fluor 555 donkey anti-mouse, or Alexa Fluor 555 donkey anti-rabbit secondary antibodies (1:400; Life Technologies, Waltham, MA, USA) for 1 h at RT in humid chamber. Cells were washed and incubated with DAPI (1:2000; Sigma-Aldrich, St. Louis, MO, USA) before mounting the coverslips.

Images were acquired using a laser confocal microscope Olympus FV1200. pATM- and Ki-67-positive cells were counted by a blinded investigator. Mean values and standard deviation were generated from at least three biological replicates.

### 4.4. Reverse Transcription and Real-Time PCR Analyses

RNA (2 μg) isolated by TRIzol (ThermoFisher Scientific) was retrotranscribed with MLV-reverse transcriptase (Promega, WI, USA) and amplified by real-time PCR using SYBR Select Master Mix (Applied Biosystem Foster City CA, USA) and gene-specific primers (Table 1). Real-time PCR was performed with the 7900HT Fast Real-Time PCR System by Applied Biosystem, Foster City, CA, USA. Relative quantification was performed by the comparative cycle threshold method. The mRNA expression values were normalized to those of the TBP (TATA-binding protein) gene, used as endogenous control. Statistical analysis was performed using Prism software (GraphPad software). Mean values and standard deviation were generated from at least three biological replicates. Statistical analysis of mRNA expression values was performed by one-way ANOVA on data normalized to the endogenous controls but not relativized in fold expression of the calibrator sample.

### 4.5. Statistical Analysis

Data are expressed as mean ± SD as indicated in figure legends. Significance was calculated using a two-tailed t test (for immunofluorescence experiments) or one-way ANOVA(for real-time PCR). *p* values of <0.05 were considered as significant in all tests.

## Figures and Tables

**Table 1:** Real-time PCR primer sequences.

Gene Name	Primer Forward	Primer Reverse
*TBP*	TGCCCGAAACGCCGAATATAATC	TGGTTCGTGGCTCTCTTATCCTC
*LMNB1*	GAAGAAGCAGCTGGAGTGGT	TTGGATGCTCTTGGGGTTCC
*CDKN1/B*	CCGCAACCAATGGATCTCCT	CAATATGGCGGTGGAAGGGA
*CCL2*	AGTCTCTGCCGCCCTTCT	GTGACTGGGGCATTGATT
*CX3CL1*	CCACCTTCTGCCATCTGACT	TTGACCCATTGCTCCTTCGG
*BTG2*	GTCTTGATGCTGCTGCCATGATC	AATAAAGGACCTGGAGTTCCCTGTAG
*COL1A2*	GGTGGGAACTTTGCTGCTCA	GCCTCTAGGTCCCATTAAGCC
*ELN*	GAGTTGGCATTTCCCCCGAA	CAAACTGGGCGGCTTTGG
*PECAM 1*	GCAACACAGTCCAGATAGTCGT	GACCTCAAACTGGGCATCAT

## Abbreviations

VEGFR	Vascular Endothelial Growth Factor Receptor
GBM	Glioblastoma
HUVECs	Human Umbilical Vein Endothelial Cells
ATM	Ataxia Telangectasia Mutated
